# Tumor-Associated Macrophages and Inflammatory Microenvironment in Gastric Cancer: Novel Translational Implications

**DOI:** 10.3390/ijms22083805

**Published:** 2021-04-07

**Authors:** Karim Rihawi, Angela Dalia Ricci, Alessandro Rizzo, Stefano Brocchi, Giovanni Marasco, Luigi Vincenzo Pastore, Fabiola Lorena Rojas Llimpe, Rita Golfieri, Matteo Renzulli

**Affiliations:** 1 Medical Oncology, IRCCS Azienda Ospedaliero-Universitaria di Bologna, 40138 Bologna, Italy; karim.rihawi@gmail.com (K.R.); dalia.ricci@gmail.com (A.D.R.); alessandrorizzo1990@virgilio.it (A.R.); fabiolalorena.rojas@aosp.bo.it (F.L.R.L.); 2 Department of Radiology, IRCCS Azienda Ospedaliero-Universitaria di Bologna, 40138 Bologna, Italy; stefano.brocchi85@gmail.com (S.B.); luigi.pastore3@studio.unibo.it (L.V.P.); rita.golfieri@unibo.it (R.G.); 3 Department of Medical and Surgical Sciences, IRCCS Azienda Ospedaliero-Universitaria di Bologna, 40138 Bologna, Italy; giovannimarasco89@gmail.com

**Keywords:** gastric cancer, tumor microenvironment, tumor-associated macrophages, tumor-associated fibroblasts, lymphocytes

## Abstract

Gastric cancer (GC) represents the fifth most frequently diagnosed cancer worldwide, with a poor prognosis in patients with advanced disease despite many improvements in systemic treatments in the last decade. In fact, GC has shown resistance to several treatment options, and thus, notable efforts have been focused on the research and identification of novel therapeutic targets in this setting. The tumor microenvironment (TME) has emerged as a potential therapeutic target in several malignancies including GC, due to its pivotal role in cancer progression and drug resistance. Therefore, several agents and therapeutic strategies targeting the TME are currently under assessment in both preclinical and clinical studies. The present study provides an overview of available evidence of the inflammatory TME in GC, highlighting different types of tumor-associated cells and implications for future therapeutic strategies.

## 1. Introduction

Gastric cancer (GC) is the fifth most diagnosed malignancy worldwide and represents the third leading cause of cancer deaths worldwide [1], despite differences in incidence and mortality among East Asia, Eastern Europe, and South America [2]. Immunotherapy has drastically improved the treatment options for many solid tumors; in advanced GC, the combination of chemotherapy and immunotherapy seems the most promising approach, based on the results of the CheckMate 649 study presented at the last European Society of Medical Oncology (ESMO) 2020 virtual meeting [3,4]. Nevertheless, the prognosis of GC patients remains dismal at present, since more than half of the patients are diagnosed at an advanced and/or metastatic stage of the disease, with a median overall survival time of roughly 1 year [2,5,6]. 

GC has traditionally been classified into two major histological subtypes, intestinal and diffuse, according to Lauren’s criteria, while the World Health Organization developed a more complex histological classification for GC, including many subtypes, some of which are very uncommon [7,8]. The main limitation of these histological classifications relies on the inability to successfully drive to histologic subtype-specific therapeutic approaches. In 2014, the Cancer Genome Atlas (TCGA) Network proposed a pioneering molecular classification, which not only provides a clearer definition of the GC biology but also has therapeutic implications [9,10,11]; according to this classification, GC encompasses four different molecular subtypes: Epstein–Barr virus-positive (9%), microsatellite instable (22%), genomically stable (20%), and chromosomal unstable (50%) [9].

On the other hand, understanding the different interactions between tumor cells, chronic inflammation, and the immune tumor microenvironment (TME) represents an unsolved clinical issue necessary for identifying and developing tailored therapies for GC, particularly for non-responder patients [12]. The TME consists of different types of cells, including immune and inflammatory cells such as lymphocytes and macrophages; stromal cells such as fibroblasts, adipocytes, and pericytes; small organelles; RNA; blood vessels and lymph vessels; the extracellular matrix (ECM), and secreted proteins. The persistent inflammatory state induced by *Helicobacter Pylori* (HP) infection and the affected gastric secretory function is supposed to play a crucial role in the transformation from chronic atrophic gastritis to metaplasia, epithelial dysplasia, and, eventually, adenocarcinoma [13]. There is also emerging evidence of several cell types involved in the GC inflammatory microenvironment, including macrophages, lymphocytes, fibroblasts, and myeloid-derived suppressor cells [14,15], as well as their products (cytokines), which may promote tumor development at the initiation, progression, and metastasis phases [14,16]. In this scenario, the presence of tumor-associated macrophages (TAMs) is attracting attention because of its promising role in predicting prognosis and drug resistance. Finally, in the era of immunotherapy, the research of TAM-targeted therapy could represent an innovative approach to tailor-made treatments and to overcome resistance to immune checkpoint inhibitors (ICIs) [16,17].

This review summarizes and discusses the current understanding of the inflammatory TME in GC, focusing on the different types of tumor-associated cells and implications for future therapeutic strategies.

## 2. Tumor-Associated Macrophages

Being a key component of tumor-infiltrating immune cells, macrophages play a crucial role in tumorigenesis; as a matter of fact, they promote tumor growth and progression and they are also involved in the development of treatment resistance [18]. Resident macrophages and recruited circulating monocytes result in TAM accumulation in the tumor mass, which generally correlates with poor prognosis across solid tumors [19]. Macrophages were initially supposed to enhance antitumor immunity, taking part in innate host defense, recognizing non-self cells, and phagocyting them [20,21]. Nevertheless, increasing preclinical and clinical evidence has highlighted the controversial role of macrophages in promoting cancer progression and metastatization [16]. Macrophages display a continuum of activation states, which is influenced by the surrounding microenvironment [22,23]. Of note, recruited monocytes can turn into non-polarized (M0) macrophages that differentiate into two distinct types of macrophages: “classically activated” or M1, which guide acute inflammatory responses, and “alternatively activated” or M2, which are involved in tumor progression [24,25]. M1 macrophages contribute to amplify the type 1 immune response through the production of proinflammatory interleukins (IL-12, IL-1, IL-6), tumor necrosis factor (TNF)-α, reactive oxygen species (ROS), and nitric oxide (NO), which are found to cause epigenetic changes in the gastric epithelial cells during HP infection [26,27]. Conversely, M2 macrophages are stimulated by the type 2 T helper cell (Th2) cytokines (IL-4, IL-10, and IL-13) and exhibit pro-tumorigenic functions, including tissue remodeling and tumor progression [28,29].

However, M2 macrophages are the most represented TAMs in the TME [30]. Preclinical evidence showed that tumor and stroma cells release C–C motif chemokine ligand 2 (CCL2) to recruit monocytes expressing C-C chemokine receptor type 2 (CCR2); these recruited CCR2-expressing monocytes will finally polarize into TAMs, contributing to tumor cell survival [31,32,33]. Thus, there is direct crosstalk between tumor cells and TAMs. Notably, tumor cells can promote M2 polarization by expressing cytokines and growth factors including colony-stimulating factor 1 (CSF-1) and IL-4 [23,34,35]. Conversely, TAMs can secrete chemokines and cytokines, particularly IL-6, IL-8, and IL-10, that are involved in tumor growth, angiogenesis, tumor invasion, and the depression of immunity [36]. Another fascinating function of TAMs in tumor progression is represented by their contribution to the formation of pre-metastatic niches (PMNs) [37]. PMNs constitute a peculiar tumor-growth-favoring microenvironment deprived of tumor cells, which is key to the survival and outgrowth of future metastatic disease [38]. Interestingly, TAMs are mobilized to the peripheral blood and then recruited in the PMNs by many tumor-secreted cytokines and growth factors (e.g., CCL2, CSF-1, VEGF, PLGF, TNF-α, TGF-β), tissue inhibitor of metallopeptidase (TIMP)-1, and exosomes [37,39,40].

Moreover, TAMs are able to profoundly modulate treatment response to chemotherapy, targeted drugs, antiangiogenic agents, immune checkpoint inhibitors (ICIs), as well as radiotherapy, resulting in treatment failure [25,41].

In GC, chronic inflammation of the gastric mucosa caused by HP infection has been established as an important risk factor in the development of the tumor, causing immune cell migration to the stomach and the production of chemokines and cytokines, which will eventually lead to the activation of survival signaling pathways and epithelial proliferation [13,42]. Unresolved inflammation derived from chronic infection with HP is one of the hallmarks of the TME in GC, which is enriched in myeloid-derived suppressor cells (MDSCs), regulatory T cells (Tregs), and TAMs [43]. In this scenario, TAMs contribute to attenuating the immune response via different pathways, including the production of anti-inflammatory cytokines (including IL-10 and TGF-β), prostaglandin E2 (PGE2), and the expression of programmed death-ligand 1 (PD-L1) [44]. In addition, TAMs play an important role in tumor neovascularization by secreting proangiogenic factors, such as VEGF, TNF, IL-1β, IL-8, PDGF, FGF, and others [45]. Finally, TAMs can also contribute to the invasiveness of tumor cells by remodeling the extracellular matrix, by expressing activated epithelial–mesenchymal transition (EMT) factor, and by promoting EMT, with invasion and migration properties [46,47].

Nevertheless, the prognostic role of TAMs in GC is still debated. In 2016, a meta-analysis including 12 studies investigated the correlation between TAM and patients’ survival, concluding that TAM infiltration and the number of infiltrating M2 macrophages might represent negative prognostic factors for GC patients [48]. In a second study by Lu et al., tumor-associated CD68+ macrophages were identified as an independent prognostic marker for recurrence-free survival (RFS), since patients with resectable GC with TAM infiltration were found to have poorer RFS [49]. Conversely, a recent study by Liu et al. demonstrated that M2 macrophage infiltration was found to be lower in signet ring cell carcinoma and mucinous adenocarcinoma, suggesting a potential positive prognostic factor [50]. Lastly, GC patients with peritoneal metastasis had increased levels of macrophages and alternatively activated macrophages in the peritoneum compared with those without peritoneal carcinomatosis [51].

A thorough understanding of the role of macrophages in tumor progression has led to the development of new, tailored therapeutic approaches. To date, macrophage-targeting strategies encompass a wide range of antibodies and small-molecule inhibitors [52]. Notably, the most important treatment strategy is represented by the inhibition of the colony-stimulating factor 1 (CSF1)–CSF1 receptor (CSF1R) axis, which is essential for macrophage survival and the transition from TAM M1 into TAM M2-type [53]. The CSF1R belongs to the platelet-derived growth factor family and, when it binds to its ligand CSF-1, it promotes myeloid differentiation, monocytic commitment, as well as the survival, proliferation, and recruitment of macrophages [16,53]. Furthermore, preclinical evidence showed that CSF1R blockade enhanced PD-1 and CTLA4 inhibitors’ activity, where ICIs showed limited efficacy [54].

Several monoclonal antibodies and tyrosine kinase inhibitors targeting CSF-1/CSF1R are being currently investigated as monotherapies or in combination in clinical trials and are summarized in Table 1.

## 3. Lymphocytes

Tumor-infiltrating lymphocytes (TILs) encompass a heterogeneous group of immune cells including B cells, T cells, and natural killer (NK) cells [55]. Among the biological mechanisms involved in anticancer immunity, T-cell adaptive immunity has been suggested to play a key role in this setting [56]; more specifically, CD8+ T cells, CD4+ T helper cells, FOXP3+ regulatory T cells, memory T cells, and NK cells can infiltrate the stromal tissue, thus modifying the host immune response against cancer cells [57,58]. Over the last decade, several reports have highlighted that the GC microenvironment could be associated with the infiltration of tumor lymphocytes [5,59]. TILs may cause potent anticancer responses, preventing tumor growth and eradicating cancer cells; moreover, TILs have been suggested to enhance inflammation through the secretion of chemokines, cytokines, and matrix metalloproteinases [60,61].

Because of the antitumor activity of T cells, patients with GC and high TILs have a better prognosis; conversely, PD-L1 expression seems to correlate with the more aggressive behavior of the disease [62,63]. In addition, a poorer prognosis was observed in GC patients showing high infiltration of FOXP3+ regulatory T cells [64,65]. In a landmark study conducted by Lee and colleagues encompassing 220 GC patients, the authors focused on the type and density of TILs and their respective roles in predicting the clinical outcome of GC [66]. The results showed that GC patients with CD3, CD4, and CD45RO TILs had prolonged survival, suggesting that TIL density could represent a predictor of regional lymph node involvement and survival in these patients. Furthermore, preclinical data have suggested that the presence of TILs may promote apoptosis in GC models, and thus, adaptive antitumor responses could be started in the GC microenvironment [27].

However, little information is available on the role of lymphocytes in GC preclinical models so far, and further efforts are needed to gain more insight into this promising, yet poorly studied, topic of GC management [67,68].

## 4. Cancer-Associated Fibroblasts

**Figure 1:**
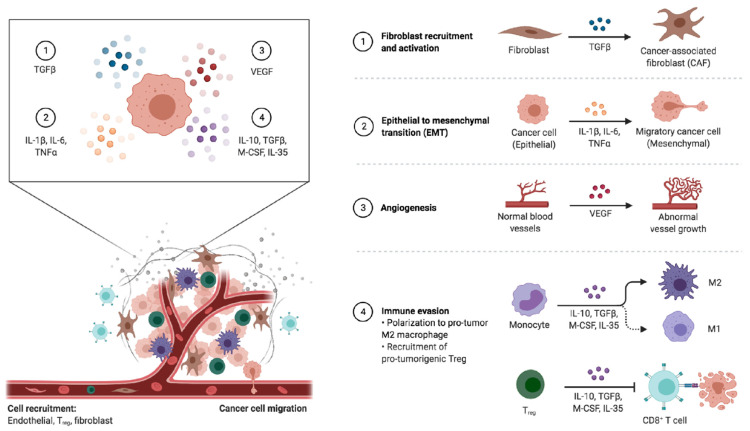
Schematic representation of tumor-associated changes observed in the tumor microenvironment. As reported below, the tumor microenvironment (TME) encompasses both the acellular elements including blood vessels and the extracellular matrix and cellular components such as epithelial cells, immune cells, and fibroblasts. The release of specific molecules by tumor cells has been suggested to modify the TME, enhancing neoangiogenesis, immune evasion, metastasis niche formation, and other processes characterizing cancer growth and progression. Abbreviations: CAF: cancer-associated fibroblast; EMT: epithelial–mesenchymal transition; IL-1 β: interleukin 1 beta; IL-6: interleukin 6; IL-10: interleukin 10; IL-35: interleukin 35; M1: M1-type macrophage; M2: M2-type macrophage; M-CSF: macrophage colony-stimulating factor; TGF-β: tumor growth factor beta; T reg: regulatory T cells; VEGF: vascular endothelial growth factor.

**Figure 2:**
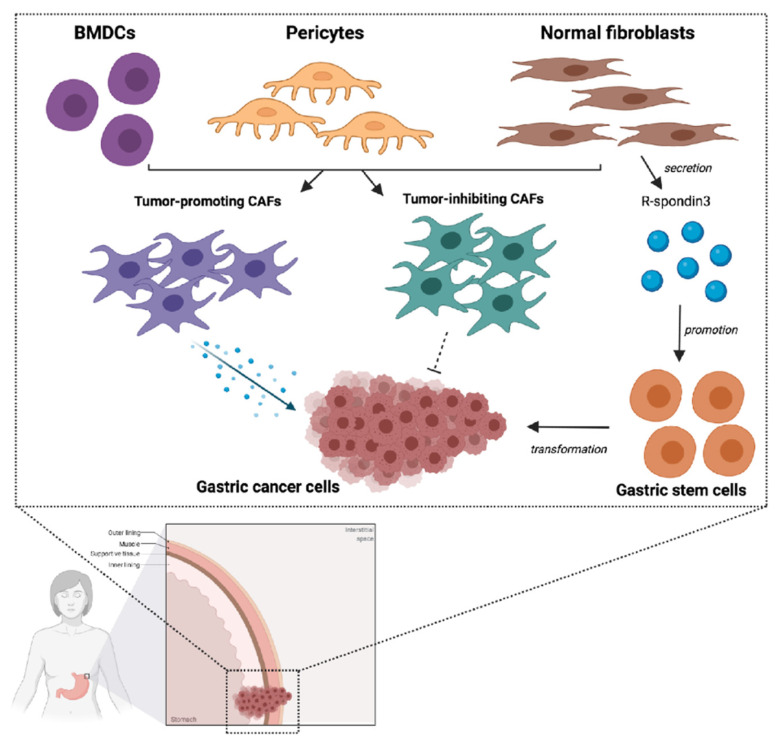
Schematic figure representing the interplay between cancer-associated fibroblasts (CAFs) and gastric cancer (GC) cells. As reported in the text, several cells represent sources of CAFs, including bone-marrow-derived cells (BMDCs), local pericytes, and normal fibroblasts, with the latter supporting GC stem cells through the secretion of R-spondin3. CAFs are able to regulate tumor growth and progression through the secretion of several molecules—such as IL-6, CXCL-12, PDGF, EGF, and FGF. Vascular endothelial growth factor A (VEGFA), CXCL12, fibroblast growth factor 2 (FGF2), and platelet-derived growth factor (PDGF) produced by CAFs facilitate the formation of new blood vessels in the TME. CAFs secrete also many chemokines and cytokines, such as CXC chemokine ligand 12 (CXCL12) and transforming growth factor-β (TGFβ), inducing immunosuppression in the TME. Notably enough, a subpopulation of CAFs has been suggested to exert also an inhibitory action on tumor cells. Abbreviations: BMDCs: bone-marrow-derived cells; CAF: cancer-associated fibroblast.

Among the numerous types of cells involved in the tumor microenvironment, cancer-associated fibroblasts (CAFs) play a key role in influencing tumor cell growth and migration (Figure 1) [69]. Conversely, these fibroblasts may also have an inhibitory action, as suggested by recent studies (Figure 2) [70,71]. Based on these premises, although targeting CAFs may represent a promising and emerging therapeutic strategy, their inhibition could also result in cancer-promoting effects.

In solid tumors rich in connective tissue, such as pancreatic cancer, CAFs have been described as large, spindle-like stromal cells [72]; interestingly, stromal tissue in physiological conditions presents a limited number of fibroblasts, and, over the last decade, genomic studies have reported the differences existing between CAFs and non-cancer fibroblasts in several solid tumors. In particular, the interaction between cancer and stromal tissue may result in the activation of CAFs [73]; when activated, these cells are able to produce soluble molecules, such as TGF-β, fibroblast growth factor (bFGF), platelet-derived growth factor (PDGF), interleukins, and molecules of the vascular endothelial growth factor (VEGF) family [74]. All these elements play a crucial role in regulating tumor growth and phlogistic responses through different systems and present a higher proliferation rate compared with normal fibroblasts [75].

The role of normal fibroblasts in the tumor microenvironment is controversial and still largely debated, with the main topic of discussion involving their functions in influencing the metastasis process [76,77]; a study conducted by Xu and colleagues aimed at replicating the environment of cancer metastasis by coculturing normal fibroblasts in monolayers of GC cells [78]. The authors observed that some tumor cells developed spindle-like morphological features and showed an increased proliferation rate as well a higher invasive potential [78]; in addition, the loss of vimentin and the E-cadherin was observed in such cells, suggesting that normal fibroblasts are able to induce EMT in tumor cells, thus leading to cancer metastasis [78]. Based on these data, normal fibroblasts are deemed able to switch their phenotype, showing CAF-like features when these cells grow in the GC microenvironment [14,79].

GCs have been frequently associated with abundant fibrosis, especially in the case of undifferentiated or poorly differentiated forms [34,80]. A study by Quante and colleagues observed an association between CAFs and GC progression and cell growth in murine models, due to the secretion of several factors—including, among others, CXCL12, IL-6, and Wnt5a [81]. These findings have been further confirmed by more recent reports, depicting specific roles for FGF, IL-6, and CXCL-12 in promoting tumor cell proliferation, and PDGF and IL-1 beta in stimulating epithelial–mesenchymal transition (EMT) (Figure 2) [82,83].

The origin of CAFs themselves is highly discussed in GC: it has been suggested that they derive from several cells, including pericytes, local physiological fibroblasts, and bone marrow mesenchymal cells (MSCs) [84,85]. According to recent reports, normal myofibroblasts may play an important role in originating CAFs [86,87]. Indeed, these cells seem to be involved in the secretion of R-spondin3, taking part in stem cell activity; moreover, during the process of mucosal inflammation and regeneration, myofibroblasts have been observed to promote proliferation and stem cell function [87,88]. Likewise, studies on GC murine models have highlighted that nearly one-fifth of the overall number of CAFs could originate from MSCs derived from bone marrow [89,90]. These findings have also been reported in patients with GCs and/or rectal adenomas following bone marrow transplantation [33,91]. Lastly, the gastrointestinal mucosa could represent another source of CAFs in GC, due to the presence of MSC-like cell types able to express Gremlin-1 [92].

The interaction between CAFs and tumor cells represents a fundamental process that is currently being investigated, with the aim of developing specific inhibitors in this setting. As previously mentioned, CAFs secrete IL-6, which has an important role in inflammatory as well as immune responses; in particular, the secretion of IL-6 is enhanced in HP-related GC [93,94]. In fact, the induction of the cyclooxygenase-2 (COX-2)/prostaglandin E2 cascade induced by HP infection leads to the hypermethylation of miR-149 in CAFs, eventually resulting in increased IL-6 secretion [94]. Based on these premises and given the role played by IL-6 in inducing EMT through the JAK2/STAT3 pathway, recent studies have investigated the role of neutralizing antibodies, including AG490 [95,96]. A study conducted by Wu and colleagues has suggested that the simultaneous inhibition of JAK2/STAT3 and IL-6 using AG490 might impair tumor diffusion induced by CAFs to the peritoneum; as such, IL-6-targeted therapies could represent a complementary treatment approach due to their action on fibroblasts [97].

CAFs are also involved in the expression of several other molecules, including VEGF, FGF, CXCL-12, and HGF [98,99]. HGF is able to induce the activation of MET tyrosine kinase, and HGF/MET has been suggested to be involved in oncogenesis and disease progression in GC [100]. Similar to IL-6, HGF inhibitors are currently being tested, with some of these agents already demonstrating encouraging results in inhibiting the transition of physiological fibroblasts into CAFs, both in vitro and in vivo [101]. In addition, the role of CAFs as a mechanism of resistance to fluoropyrimidines in GC patients is also being studied, with preliminary reports suggesting that complementary treatments inhibiting CAFs may overcome resistance to 5-fluorouracil [102,103].

Lastly, other promising therapeutic options include triptolide and tranilast [104]. Triptolide is a diterpenoid triepoxide derived from the herb Tripterygium wilfordii, which has been used as a natural agent; preclinical studies have suggested that triptolide treatment may inhibit the migration-, formation-, and invasion-promoting action of CAFs in GC [104]; in addition, this agent has been shown to downregulate microRNA-301a expression, while upregulating microRNA-149 expression in CAFs, a process leading to increased production of tumor-suppressive factors and to the inhibition of the secretion of the oncogenic IL-6. Finally, triptolide may impair the induction of EMT in GC cells by CAFs. Tranilast (N-[3,4-dimethoxycinnamoyl]-anthranilic acid) inhibits the release of chemical mediators from mast cells and it is used as an anti-allergic agent clinically; in oncology, it has shown an inhibitory action towards the interaction between GC cells and CAFs, through the inhibition of GC growth and fibrosis; thus, tranilast has been suggested to prevent peritoneal dissemination in GC preclinical models [105].

Currently, a large number of potentially meaningful clinical targets is under assessment in GC patients with CAFs inhibition, surely being a novel and promising anticancer strategy; however, investigational CAF-targeted therapies need further validation, and effective drugs against CAFs are currently lacking [105,106]. The results of ongoing trials are eagerly awaited in this setting.

## 5. Mesenchymal Stem Cells

As schematically reported in Figure 1, mesenchymal stem cells (MSCs) within the tumor microenvironment are able to differentiate into CAFs and tumor-associated MSCs, following their migration to tumors [107,108]. From a molecular point of view, MSCs play a stimulating action towards the angiogenetic process, due to the secretion of FGF, PDGF, and VEGF [109,110]; in fact, MSCs enhance the metastatic potential of tumor cells and favor drug resistance. Based on their potential role, MSCs are being investigated as therapeutic targets in solid tumors; specifically, MSCs might act as drug vehicles, given their marked tendency to be home to cancer sites (Table 2) [111,112]. For example, a phase I/II study evaluated the role of MSCs in gastrointestinal malignancies, including GC (NCT02008539); the trial assessed the role of modified MSCs combined with ganciclovir, reporting promising results in terms of both safety and efficacy [113].

Another therapeutic strategy including modified MSCs is based on the expression of cytosine deaminase and the co-administration of 5-fluorocytosine; such a combination was shown to inhibit gastrointestinal cancer cells’ growth in immunocompromised murine models [114,115,116]. In addition, apoptosis-inducing factors are also being evaluated within phase I and II studies exploring the role of oncolytic measles virus encoding thyroidal sodium iodide symporter (MV-NIS)-infected MSCs (NCT02068794); however, further efforts are needed to clarify whether MSCs could enter into clinical practice, confirming their potential in GC therapy.

## 6. Immunotherapy and TME in Gastric Cancer

Pathways regulating the immune system are called immune checkpoints: different cancer types use these immune checkpoints as a mechanism of immune evasion, leading to tumor progression [117,118]. The dependence of cancer on these pathways has been therapeutically exploited with the development of specific immune checkpoint inhibitors (ICI) such as anti-programmed cell death 1 (anti-PD-1) and anti-PD-1 ligand (anti-PD-L1) antibodies. By blocking the PD-1 axis with anti-PD-1 or anti-PD-L1 monoclonal antibodies, anti-tumor immune responses can be restored, leading to tumor regression. Briefly, PD-1 is a negative co-stimulatory transmembrane protein expressed on T-cells, B-cells, and NK cells; its binding to PD-L1 (which is expressed on tumor cells) and PD-L2 leads to peripheral T effector cell modulation as well as to tumor cell apoptosis and increased conversion of T effector cells to Treg cells [118].

In GC, pembrolizumab and nivolumab (both anti-PD-1 antibodies) have demonstrated survival benefit and gained regulatory approval in advanced settings; namely, pembrolizumab received accelerated approval for treatment of PD-L1-positive GC in third-line or later treatment by the US Food and Drug Administration (FDA) and for the treatment of patients with unresectable or metastatic, microsatellite instability-high (MSI-H), or mismatch repair-deficient (MMR-D) solid tumors that have progressed following prior treatment [118].

TME and stromal extracellular matrix (ECM) proteins can have a immune-modulatory functions, thus contributing to the immune evasion of cancer cells [119,120]. Immune surveillance is able to monitor, detect, and destroy cancer cells; however, as tumor progresses, cancer cells develop various mechanisms of immune evasion, leading to the development and the invasiveness of the tumor. A highly immunosuppressive microenvironment has been recently associated with the cross-talk between non-tumor-cell mesenchymal stromal cells (MSCs) and TAMs [121]. Namely, the differentiation from myeloid cells into M2-polarized macrophages, which, as mentioned before, are extremely immunosuppressive, seems to be mainly driven by exosomes produced by MSCs. Moreover, the interactions between pro-inflammatory macrophages and MSCs, mainly mediated by CD54, can lead to an increase in the immunosuppressive activity of MSCs [121]. As such, the blockade of such a network between MSCs and TAMs may represent a new therapeutic target to exploit in order to restore immune tolerance and improve clinical outcomes in GC patients. 

Finally, although immunotherapy can result in long-lasting and major responses, a large portion of patients, however, do not benefit from this therapeutic approach, either from the beginning (i.e., primary resistance) or progressing after an initial response (i.e., acquired resistance). TAMs can limit the efficacy of immunotherapies, playing a tumor-supportive role; moreover, they can also reprogram MSCs to a state which is ideal for immunotherapy resistance in the tumor niche [120]. Interactions between MSCs and TAMs are complex and may represent a promising target for immunotherapy; however, the molecular and cellular mechanisms underlying these interactions have not been thoroughly identified and further evidence is still warranted.

## 7. Conclusions

The TME represents a “complex society” composed of a wide spectrum of cell types and their extracellular matrix, where inflammation acts as primum movens in creating the TME and gastric carcinogenesis. This review provides an overview of available evidence of the inflammatory TME in gastric cancer, highlighting different types of tumor-associated cells and implications for future therapeutic strategies. As of today, several agents and therapeutic strategies targeting the TME are currently under assessment in both preclinical and clinical studies. Understanding the precise biology of the tumor microenvironment is a matter of topical and urgent importance and several preclinical and clinical strategies are currently under evaluation to target subpopulations of specific cells in this setting. Besides the progress in molecular and cellular biology, an improved understanding of the tumor microenvironment as a whole, with its complex network of cells and cytokines, will contribute to the development of promising new therapeutic approaches in the treatment of GC.

## Figures and Tables

**Table 1:** Clinical trials targeting tumor-associated macrophages in solid tumors, including gastric cancer.

Drug	Target	Class of Drug	Mechanism of Action	Clinical Trials Identifier	Planned Enrollment	Design	Drug Combined with
**Emactuzumab (RG7155)**	CSF1R	mAB	Directed against CSF1R expressed on macrophages, interferring with the CSF-1/CSF1R axis	NCT02323191 (completed)	221 pts	Phase I	Atezolizumab
				NCT02760797 (completed)	38 pts	Phase I	Selicrelumab
				NCT01494688 (completed)	217 pts	Phase I	Paclitaxel
**Cabiralizumab (FPA008)**	CSF1R	mAB	Binds to to CSF1R and blocks its signaling, reducing TAMs and promoting a proinflammatory microenvironment and T-cell responses	NCT03335540 (recruiting)	50 pts	Phase I	Nivolumab
				NCT03431948 (active, not recruiting)	60 pts	Phase I	Nivolumab + SBRT
				NCT02526017 (completed)	295 pts	Phase I	Nivolumab
				NCT03158272 (completed)	19 pts	Phase I	Nivolumab
**IMC-CS4**	-	mAB	Directed against CSF1R, which may trigger ADCC in tumor cells overexpressing CSF1R.	NCT01346358 (completed)	72 pts	Phase I	none
**ARRY-382**	CSF1R	TKI	Orally administered, small-molecule macrophage CSFR1R antagonist	NCT02880371 (completed)	82 pts	Phase I-II	+/- Pembrolizumab
				NCT01316822 (completed)	26 pts	Phase I	none
**BLZ945**	CSF1R	TKI	Selectively binds to CSF1R expressed on TAMs, blocks the activity of CSF1R, and inhibits CSF1R-mediated signal transduction pathways.	NCT02829723 (recruiting)	200 pts	Phase I	none
**PLX7486**	CSF1R, TrkA, TrkB, TrkC	TKI	Selective inhibitor of CSF1R and TrkA, TrkB, and TrkC.	NCT01804530 (completed)	59 pts	Phase I	none

CSF1R: colony-stimulating factor 1 receptor; mAB: monoclonal antibody; pts participants; SBRT: Stereotactic Body Radiation Therapy; ADCC: antibody-dependent cell-mediated cytotoxicity; TKI: tyrosine kinase inhibitors; TAMs: tumor-associated macrophages; TrkA, TrkB, and TrkC neurotrophic tyrosine kinase receptor types 1, 2, and 3.

**Table 2:** Clinical trials evaluating transplantation of mesenchymal stem cells in advanced solid tumors, including gastric cancer.

NCT Name	Phase	Setting	Treatment	Recruitment Status	Estimated Enrolment	Primary Outcomes
NCT02008539	I/II	Advanced gastrointestinal malignancies	MSC_apceth_101	Terminated	13	Safety
NCT02068794	I/II	Relapsed fallopian tube, peritoneal, or ovarian cancer	Oncolytic measles virus encoding thyroidal sodium iodide symporter (MV-NIS)-infected mesenchymal stem cells	Recruiting	57	MTD
NCT03298763	I/II	Advanced non-small-cell lung cancer	Targeted stem cells expressing TRAIL	Recruiting	46	RP2D Tumor response rate

RP2D: recommended Phase II dose; MTD: maximum tolerated dose.

## Data Availability

No new data were created or analyzed in this study. Data sharing is not applicable to this review.
